# Effects of Quercetin on the Intestinal Microflora of Freshwater Dark Sleeper *Odontobutis potamophila*

**DOI:** 10.3390/antiox11102015

**Published:** 2022-10-12

**Authors:** Chenxi Zhu, Guoxing Liu, Xiankun Gu, Tongqing Zhang, Aijun Xia, You Zheng, Jiawen Yin, Mingming Han, Qichen Jiang

**Affiliations:** 1 Freshwater Fisheries Research Institute of Jiangsu Province, Nanjing 210017, China; 2 Geography Section, School of Humanities, Universiti Sains Malaysia, Minden 11800, Malaysia; 3 College of Animal Science and Technology, Yangzhou University, Yangzhou 225009, China; 4 Biology Program, School of Distance Education, Universiti Sains Malaysia, Minden 11800, Malaysia

**Keywords:** quercetin, odontobutis potamophila, intestinal microflora

## Abstract

Flavonoids have antimicrobial and anti-oxidation properties. The effects of the flavonoid quercetin on the intestinal microflora of freshwater dark sleeper *Odontobutis potamophila* were tested for the first time. *Odontobutis potamophila* juveniles were treated with quercetin for 21 days at one of three concentrations (2.5, 5.0, or 10.0 mg/L) and compared with a control group that was not treated with quercetin. Quercetin improved the stability of the intestinal flora in *O. potamophila* and the probiotic bacteria *Bacillus* spp. and *Lactobacillus* spp. increased in species abundance after the low concentration quercetin treatments. Furthermore, the abundance of pathogenic bacteria *Plesiomonas* spp., *Aeromonas* spp., and *Shewanella* spp. decreased after the fish had been exposed to quercetin. Activity of hepatic antioxidant enzymes (superoxide dismutase, SOD), (glutathione S-transferase, GST), (glutathione peroxidase, GSH-Px), and (total antioxidant capacity, T-AOC) increased in the livers of *O. potamophila* treated with quercetin, thereby increasing their hepatic antioxidant capacity and their ability to scavenge free radicals.

## 1. Introduction

Aquaculture has expanded significantly in recent years and fish are now one of the main sources of protein consumed by humans. As fish farming has become more intensive, the risk of infectious disease outbreaks on fish farms has substantially increased [1]. However, the use of antibiotics as fish feed additives has a detrimental effect on fish growth, causes oxidative stress, and affects their histopathology [2,3]. More importantly, antibiotics thus accumulate in the food chain. Furthermore, the spread of aquatic antibiotic-resistant bacteria impacts human health. Indeed, it is conservatively estimated that antibiotic-resistant infections kill about 23,000 people a year in the United States [4]. Therefore, in 1997, the European Union banned antibiotics from farm feed [5].

Alternatives to antibiotic feed additives are economically critical to sustainable aquaculture and the provision of safe and nutritious fish products for human consumption. Quercetin is a flavonoid compound that is widely distributed in foods and vegetables, such as tea [6], apples [7], onions [8], milk thistle [9], and red wine [10]. It has been demonstrated that flavanonol has advantageous biological impacts on health, such as anti-inflammatory [11], antimicrobial, antituberculosis [12], hepatoprotective [13], cardiovascular [14], anti-angiogenic [15], and anticancer [16] activities. The above functions of quercetin have been verified in some model animals, such as *Caenorhabditis elegans* [17], mice [18], and humans [19]. The anti-inflammatory and antioxidant properties of quercetin suggest that it could potentially be an alternative to antibiotic feed additives. The antioxidant properties of quercetin in *O. potamophila* have been demonstrated by previous studies in early research [20], but there is still a lack of information about some of the effects of quercetin on the gut microbiology of aquatic organisms.

Numerous studies have revealed that host lipid metabolism, insulin sensitivity, systemic inflammation, and energy homeostasis may all be affected by changes to the composition of gut microflora [21,22,23] found that flavonoids have an antimicrobial effect by inhibiting nucleic acid synthesis by microbes. The antibacterial components of bioactive flavonoids also interact with the hydrophilic region of phospholipids on the cell membrane and eventually penetrate the hydrophobic core when the concentration of flavonoids increases. Quercetin reverses imbalances in intestinal microbiota and the associated dysbiosis-mediated induction of the Toll-like receptor 4 (TLR-4)-NF-κB pathway in mice fed on a high-fat diet. It subsequently inhibits the inflammasome response and activation of reticulostriatal pathway, and increases the expression of genes associated with lipid metabolism [24]. Plant-based feed additives such as quercetin affect several biological parameters. For example, they increase the secretion of digestive enzymes, change immune responses, and increase nutrient absorption, all of which eventually result in a better growth performance by the animals [25]. However, half of the studies we evaluated failed to specify whether intestinal microflora influence biological parameters.

The commercially valuable freshwater fish known as the “dark sleeper”, *Odontobutis potamophila* [26], is widespread in the river systems of China and Southeast Asian nations. This species has a high meat content, strong flavor, and high nutritional value. Its potential profitability makes it an attractive candidate for aquaculture [27]. Therefore, this study provides fresh insights into the effects of quercetin on intestinal microbial diversity by studying the effects of quercetin on fish feed supplementation.

The objective of this study was to analyze how long-term exposure to quercetin may impact the bacterial communities in *O. potamophila*, its immune response and their potential interaction. Illumina next-generation sequencing was used to examine alterations to the gut microbiomes. The results show the bacterial phylotypes that were affected and the potential functional impacts. These findings provide reference information that can be used to increase our knowledge about the impact of quercetin on freshwater species.

## 2. Materials and Methods

### 2.1. Animal Culture

The Freshwater Fisheries Research Institute of Jiangsu Province, China provided the 144 *O. potamophila* samples used in this study, which weighed 1.10 ± 0.05 g. The fish were maintained for 16 days in cultured freshwater (UV-sterilized and well-aerated water; pH 7.5 ± 0.5; dissolved oxygen, 5 mg/L) under laboratory conditions at 25 ± 1 °C. During the 2 weeks’ acclimation period, all *O. potamophila* were fed daily with *Limnodrilus hoffmeisteri* at 7:00 a.m. and 8:00 p.m. The feeding rate was set at 5% of the *O. potamophila* body weight.

### 2.2. Experimental Design and Sample Collection

A pre-experiment showed that 10 mg/L quercetin was not harmful to *O. potamophila*. Quercetin (Sigma-Aldrich, St Louis, MO, USA), purity > 98%, was dissolved in Dimethyl sulfoxide before use and stored at −20 °C away from light. Both treatment and control groups contain 0.05% DMSO. In this study, the fish were maintained in an 8 L glass tank and exposed to different concentrations (2.5, 5, or 10 mg/L) of quercetin with a quercetin-free treatment as the control group. There were six replicate aquariums for each treatment and the control, and each aquarium contained six *O. potamophila*. All four treatments experienced the same culture conditions during the acclimation period. The *O. potamophila* were fed with *L. hoffmeisteri* at 7:00 a.m. and 8:00 p.m. daily for 21 days during the exposure period. The experimental solution was refreshed every day. Four experimental groups were used the current study: control (CK) and Q2.5, Q5, and Q10, indicating the exposure of *O. potamophila* to 0, 2.5, 5.0, and 10 mg/L quercetin, respectively.

After 21 days, the 144 samples were anesthetized on ice and then intestinal and hepatopancreas tissue samples were collected using sterile scissors and forceps. The removed tissues were transferred to enzyme-free centrifuge tubes and stored in liquid nitrogen.

### 2.3. Illumina Miseq Sequencing

The standard operating procedures for the Illumina MiSeq platform (Illumina, San Diego, CA, USA) were used to construct PE 2 × 300 libraries from purified and amplified fragments. The raw sequences were imported as FASTQ files into the file format so that they could be subsequently processed by QIIME2 platform. In addition to quality control measures, such as pruning, denoising, splicing, and removing chimeras, the QIIME2 DADA2 plug-in was also used to obtain a final list of features. We used the 338F/806R primer pair to compare the representative ASV sequences to those in the GREENGENES database with 99% similarity. A taxonomic information table for the species was obtained and contaminating mitochondria were removed. ANCOM, ANOVA, Kruskal–Wallis, igraph, LEfSe, and DEseq2 analyses were used to identify any bacteria that differed among the treatments and any abundance differences among the samples. These analyses were carried out by “mixOmics”, which is part of R package. A partial least squares discriminant analysis is a supervised statistical method that is used to reveal the relationship between microbiota and sample classes so that sample class predictions based on the relative abundance of major microbiota species can be made. Furthermore, we calculated Spearman rank correlation coefficients to understand the relationships between species based on a co-occurrence analysis. The parameters used in this analysis were the default settings unless otherwise noted. PICRUSt was also used to predict the likely functional composition of the microbiome.

### 2.4. Quantitative Real-Time PCR Analysis

RNA was extracted from the hepatopancreas of *O. potamophila* for real-time fluorescence qPCR analysis. The total RNA hepatopancreas tissue sample was reverse transcribed into cDNA using a PrimeScript RT reagent kit (Takara, Shiga, Japan) and stored at −80 °C for further analysis. RT-qPCR was performed using CFX96 RT-PCR (BioRad, Hercules, CA, USA) and TransStart Top Green qPCR SuperMix (TransGen, Beijing, China). Detailed information is available in the Table 1.

### 2.5. Antioxidant Enzyme Activity Assay

Each concentration group contained six individuals and was replicated six times. Samples were collected after 21 days’ exposure. All the fish in each replicate were collected, mixed, and quickly frozen in liquid nitrogen. Hepatopancreas tissue from each individual (100 mg) was homogenized in 400–800 μL PBS (pH 7.4) and centrifuged for 20 min at 16,873× *g* and 4 °C. The collected supernatant was used for enzyme activity testing. The assays for total antioxidant capacity (T-AOC), and the superoxide dismutase (SOD), peroxidase (POD), glutathione peroxidase (GSH-Px), glutathione S-transferase (GST), and glutathione (GSH) were performed using their respective reagent kits (Nanjing Jiancheng, China) according to the manufacturer’s protocol.

### 2.6. Data Analysis

The software programs GraphPad Prism 8 and SPSS 20.0 (IBM, Armonk, NY, USA) were used to analyze the data. SPSS 20.0 was also used to graphically evaluate the experimental data. The 2^−ΔΔCT^ method was used to evaluate the relative mRNA levels of the target genes and ANOVA was used to determine the significant differences (*p*-value, *p* < 0.05) between the treatment and control groups. The values are expressed as the mean + standard deviation.

## 3. Results

### 3.1. S rRNA Sequencing Data and Species Evaluation

**Figure 1:**
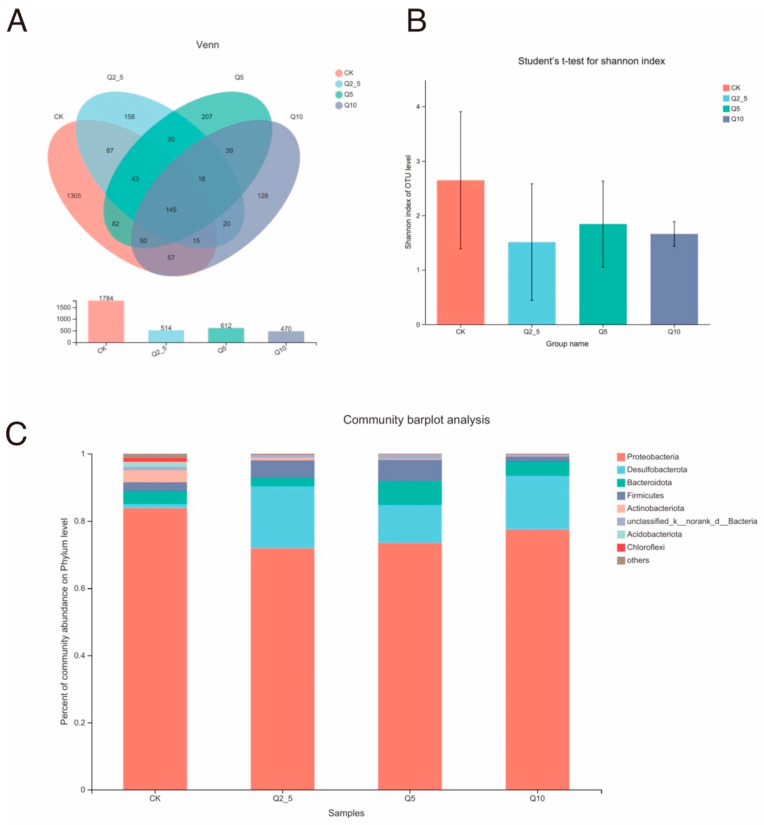
(**A**) Venn diagram showing common or endemic species. Different colors represent different groups. The numbers in the overlapping part represent the number of species common to multiple groupings and the numbers in the non-overlapping part represent the number of species unique to the corresponding grouping. (**B**) α-Diversity analysis of the gut microbial community differences between the control group and the three experimental groups. The values are presented as Shannon index values. A one-way ANOVA with Student’s *t*-test were used to determine significant differences between the control and the three experimental groups: *p* < 0.05 was considered statistically significant (* *p* < 0.05). (**C**) Histogram of the relative distribution of each group at the phylum level (species in the top nine in terms of relative abundance). The ordinate indicates the ratio of the number of sequences annotated at the phylum level compared to the total annotated data. The color order from bottom to top corresponds to the color order of the legend on the right.

The *O. potamophila* intestinal microorganisms exposed to quercetin were classified to the phylum, order, family, genus, and species taxonomic levels. After removing the low-quality reads, a diversity data analysis of 20 samples was completed and a total of 837,257 optimized sequences, with an average sequence length of 427 bp. and 2370 operational taxonomic units (OTUs) were obtained. Most of the sequence lengths met the sequencing requirements, and it was concluded that the sequencing results covered all sequences in the V3–V4 region of the 16S r RNA gene. There were 145 OTUs that were shared among the four groups. The CK, Q2.5, Q5, and Q10 groups contained 1305, 158, 207, and 128 unique OTUs, respectively (Figure 1A).

The Shannon index was used to compare the gut microbial community alpha diversities in the three experimental groups to the control group alpha diversity (Figure 1B). The alpha diversity of the gut microbial community increased with the Shannon index value. There was a decrease in intestinal microbial community diversity after quercetin treatment, but it was not significant (*p* > 0.05).

Most of the intestinal microorganisms in the samples from each group were classified into five core phyla: Proteobacteria, Desulfobacterota, Bacteroidota, Firmicutes, and Actinobacteria. After treating the fishes with different concentrations of quercetin, the distributions of the four dominant groups were similar in each sample, but the abundance and trends varied (Figure 1C). Desulfobacterota abundance increased the most after quercetin. Furthermore, 83.80% of the phyla in the CK group were Proteobacteria and 1.27% were Desulfobacteria.

### 3.2. Species Analysis at the Genus Level

**Figure 2:**
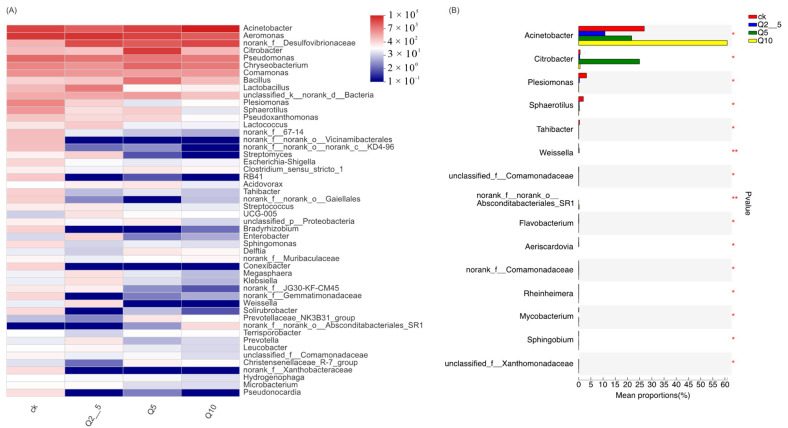
(**A**) Heat map of different genes. Heat map showing differences at the genus levels. The horizontal direction is the sample information and the vertical is the annotation information for genus and species. Red indicates a higher relative species abundance and blue indicates a lower relative species abundance. (**B**) Genes exhibiting differences in abundance among the four groups. The *Y*-axis indicates the genes name at a given taxonomic level and the *X*-axis indicates the mean relative abundance of the genes among the different groupings. The different colored bars indicate different groupings. The value on the right is the *p*-value where *p* < 0.05 was considered statistically significant (* *p* < 0.05, ** *p* < 0.01). Samples were divided into four groups: CK, Q2.5, Q5, and Q10.

**Figure 3:**
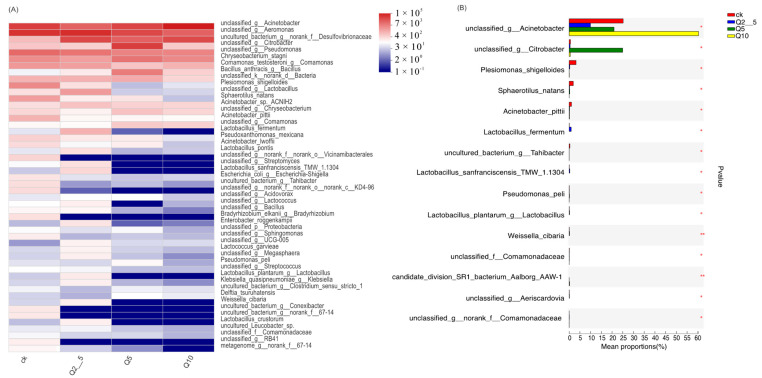
(**A**) Heat map of different species. Heat map showing differences at the genus and species levels. The horizontal direction is the sample information and the vertical is the annotation information for species. Red indicates a higher relative species abundance and blue indicates a lower relative species abundance. (**B**) Species exhibiting differences in abundance among the four groups. The *Y*-axis indicates the species name at a given taxonomic level and the *X*-axis indicates the mean relative abundance of the species among the different groupings. The different colored bars indicate different groupings. The value on the right is the *p*-value where *p* < 0.05 was considered statistically significant (* *p* < 0.05, ** *p* < 0.01). Samples were divided into four groups: CK, Q2_5, Q5, and Q10.

Figure 2A shows differences at the genus levels. Figure 2B shows genes exhibiting differences in abundance among the four groups, the *Plesiomonas, Shewanella, Weissella,* and *Rheinheimera* abundances were significantly lower than that of the control group (*p* < 0.01). Figure 3A shows differences at the species levels. In addition, Figure 3B shows that the *Plesiomonas shigelloides* and *Weissella cibaria* abundances were significantly lower than that of the control group (*p* < 0.01).

### 3.3. Analysis of the Biochemical Components

**Figure 4:**
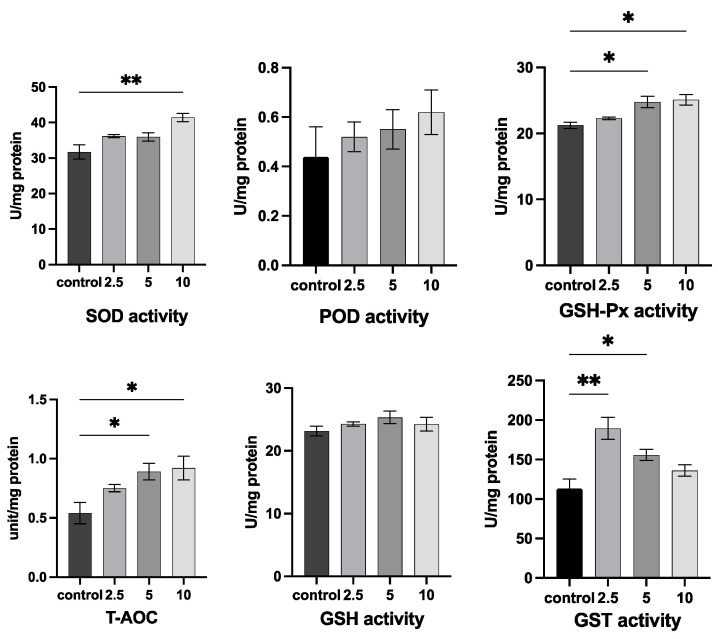
Changes in antioxidant enzymes (SOD, POD, GSH-Px, T-AOC, GSH, and GST) and T-AOC in *O. potamophila* after exposure to quercetin for 21 days. Mean ± SD (*n* = 6 for the treatment and CK group). Significantly different from control values (* *p* < 0.05, ** *p* < 0.01).

It can be seen from Figure 4 that adding quercetin to *O. potamophila* affected antioxidant enzymes (SOD, GSH-Px, T-AOC, and GST) as well as T-AOC. The higher quercetin concentrations (5 and 10 mg/L) significantly increased GSH-Px and T-AOC contents (*p* < 0.05). In addition, 10 mg/L quercetin significantly increased SOD activity (*p* < 0.01). Quercetin at 2.5 mg/L (*p* < 0.01) and 5 mg/L (*p* < 0.05) also significantly increased GST activity, while (peroxidase, POD) and (glutathione, GSH) activities were not affected by quercetin treatment.

### 3.4. Quercetin Strengthens the Immune System

**Figure 5:**
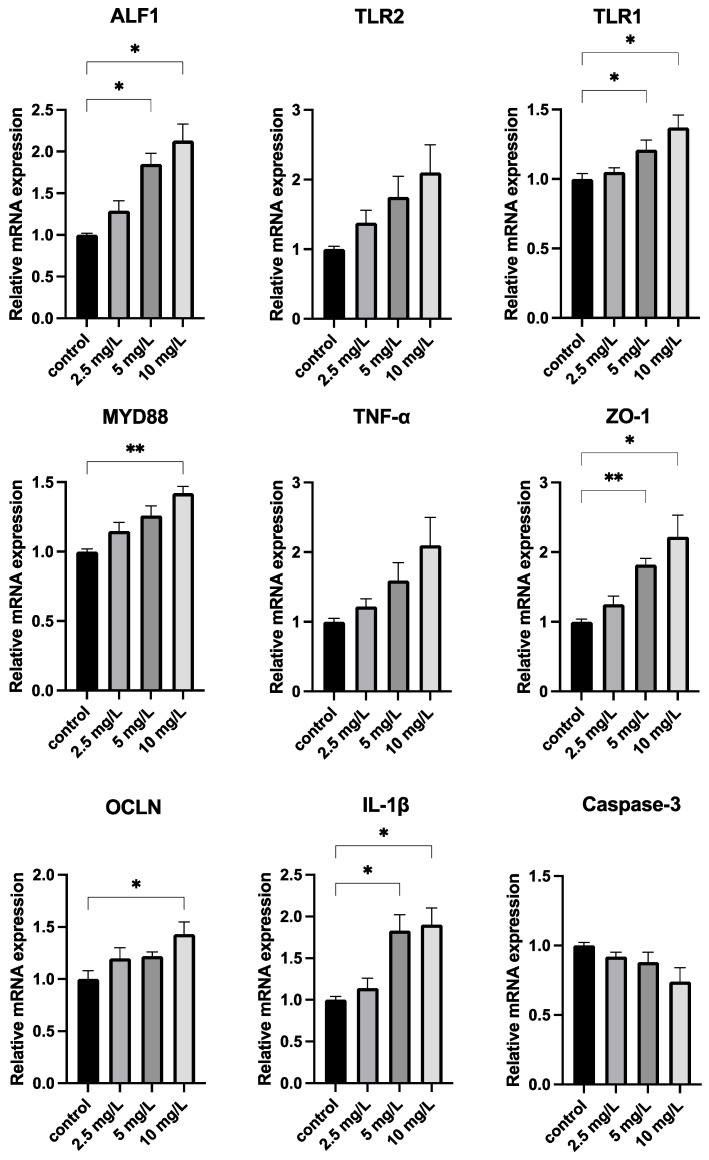
Levels of mRNA in the genes associated with the immune response in the liver of *O. potamophila* after exposure to quercetin. Values are given as mean ± SD (*n* = 3). “*” indicates *p* < 0.05 compared to the control; “**” indicates *p* < 0.01 compared to the CK.

Figure 5 shows that the relative expressions of ALF1, TLR1, and ZO-1 were significantly higher (*p* < 0.05) at Q5 and Q10 compared with the CK group. MDY88 gene expression was also significantly higher in the Q10 treatment group (*p* < 0.01) than in the CK group.

### 3.5. Linear Discriminant Analysis (Lda) Integrated with Effect Size (LEfSe)

**Figure 6:**
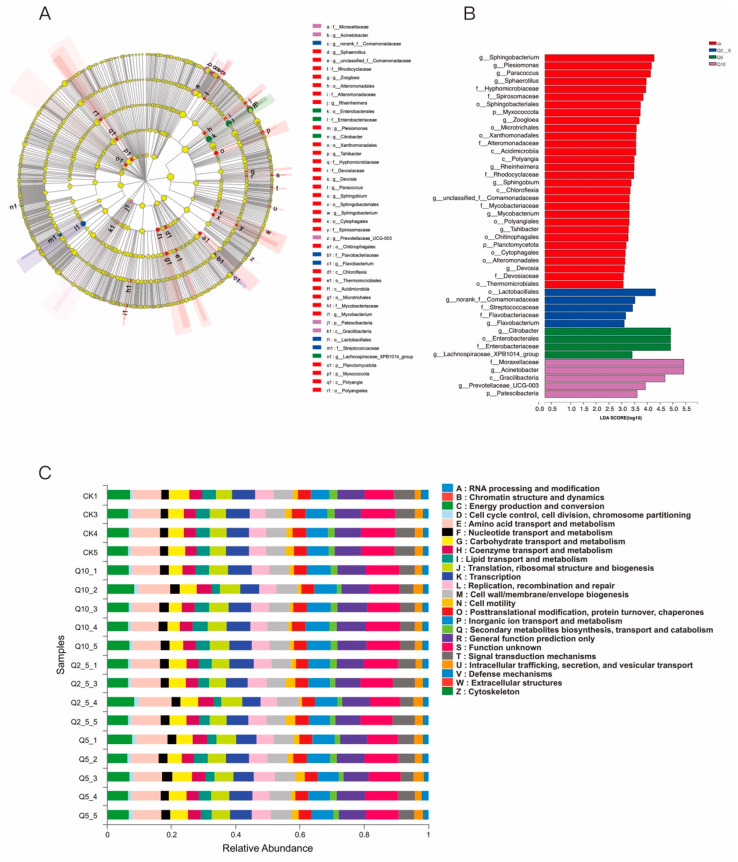
Regulation of bacterial metabolic pathways by quercetin. (**A**) LDA analysis; (**B**) LEfSe analysis; and (**C**) analysis of the KEGG metabolic pathway differences among the groups.

A LEfSe analysis was conducted to identify specific microorganisms associated with quercetin treatment to determine the differences among the intestinal bacterial communities in the four groups, (Figure 6A,B). Proteobacteria were more abundant in the control group, but the proportion of opportunistic pathogenic bacteria decreased in the quercetin-treated groups. This result suggests that quercetin maintains the structural balance of the intestinal microbiota.

We then investigated the effect of quercetin on the potential metabolic pathways of the gut microbiota in *O. potamophila* using a PICRUSt analysis and the KEGG method. Figure 6C shows that metabolism by the gut microbiota was enhanced in the quercetin-treated group. For example, the abundance of microorganisms associated with energy production and conversion and the amino acid transport and metabolism pathways increased in the quercetin-treated group.

## 4. Discussion

Microbes in the gut play an influential role in host health by breaking down nutrients, such as enzymes, amino acids, and vitamins, and by providing physiologically active substances [28]. The gut microbiota, which directly affect host digestive function and immune responses, are a key health indicator of fish health. Flavonols have a probiotic-like anti-inflammatory effect on intestinal mucosal inflammation in vitro and in vivo [29]. Polyphenols can also interfere with the bioavailability of intestinal microbiota and regulate them [30]. In this study, we used bacterial microbial diversity analysis techniques to evaluate the quercetin mechanism of action at the molecular level in *O. potamophila* and its effect on intestinal microorganisms.

The histogram (Figure 1C) showing the relative distribution of each group at the phylum level indicates that Proteobacteria, Desulfobacterota, Bacteroidota, and Firmicutes dominate the intestinal tract of *O. potamophila* regardless of diet. References [31,32] demonstrated that Proteobacteria was the most ubiquitous and common phylum, but the Bacteroidota and Firmicutes relative abundances were low. Proteobacteria are frequently seen as indicators of mammalian microbial community instability and can cause dietary and metabolic issues [33]. Inflammation is also closely related to variations in proteobacterial abundance [34]. However, quercetin significantly decreased Proteobacteria abundance and stabilized intestinal flora in this experiment.

Through increased feed conversion, improved water quality, or stimulation of the host immune system, probiotics can prevent pathogens from spreading in the gut and improve fish health [35]. In addition, probiotics reduce obesity and liver damage caused by a high-fat diet through regulation of host metabolism and gut microbiota [36,37,38]. The genera *Bacillus* and *Lactobacillus* belong to the phylum Firmicutes. Quercetin increased *Bacillus* abundance. *Bacillus* is one of the most common probiotics used in aquaculture to enhance the immune response and disease resistance [39]. Several studies have demonstrated that it has immunomodulatory effects on fish [28,40], and it is often used as an aquaculture probiotic to promote the feed absorption rate [41]. Metabolic dysregulation can eventually lead to hepatic steatosis because obesity impairs hepatic glucose and lipid homeostasis [42,43]. Quercetin is often used as a drug to regulate the expression of hepatic genes related to lipid metabolism and to prevent high-fat diet (HFD)-induced obesity [44,45]. Microorganisms, such as Bacilli and Streptomyces, produce high levels of 1-deoxynojirimycin (DNJ), which reduces insulin and glucose levels, thereby improving carbohydrate metabolism and accelerating hepatic glucose metabolism due to the inhibitory effect of DNJ on glucose intestinal absorption and the restoration of hepatic glucose and lipid homeostasis [46,47]. Overall, quercetin increases *Bacillus* abundance, increases DNJ production, and accelerates lipid metabolism by improving carbohydrate metabolism.

This study showed that *Lactobacillus* spp. abundance increased after the 2.5 mg/L quercetin treatment, but *Lactobacillus* spp. abundance decreased in the 5 mg/L and 10 mg/L quercetin treatments compared to the control group, indicating that the quercetin effect on *Lactobacillus* spp. was related to its concentration. This is consistent with the effect of quercetin on human *Lactobacillus* spp. At 4 μg/mL, *Lactobacillus* spp. abundance increased in humans, whereas 20 and 50 μg/mL quercetin led to a decrease in *Lactobacillus* spp. abundance [48], which indicates that quercetin is toxic at certain concentrations. However, further experiments on fish using different concentration gradients are needed if it is to be used as a feed additive.

The phylum Proteobacteria contains the genera *Plesiomonas, Aeromonas*, and *Shewanella*. An experiment studying growth-related gut microbes in discus fish (*Symphysodon haraldi*) [49] found that *Lactococcus* promotes fish growth, whereas *Plesiomonas* restricts it. The pathogenic bacterium *Plesiomonas shigelloides* is the only species in the genus *Plesiomonas* and is known to cause fish diseases, as in references [50,51], which studied the structure and function of the cytotoxic outer membrane protein (ComP) of *P. shigelloides* and found that ComP may contribute to host cell death.

Human patients infected with *P. shigelloides* develop cholera-like diarrhea after the consumption of contaminated water and raw seafood [52]. Furthermore, immunocompromised patients are more likely to develop several extra-intestinal diseases, such as pneumonia, sepsis, and meningitis. These infections can lead to increased mortality rates [52]. *Plesiomonas* is also the main cause of mortality in carp and salmon [53]. Quercetin treatment significantly reduced the number of *Plesiomonas*, suggesting that quercetin improves disease resistance and is beneficial to *O. potamophila* health.

*Aeromonas* spp. are potentially pathogenic to fish because they may cause skin ulcers, bacterial enteritis, and septicemia [54]. Quercetin treatment reduced the abundance of s__unclassified_g__*Aeromonas* species. There have been many studies on natural compounds that could potentially be used to treat fish diseases caused by *Aeromonas*. For example, exposure to neem (*Azadirachta indica*) oil as a nano-emulsion increased the activity of the antioxidant enzymes CAT and GST in common carp infected with *Aeromonas culicicola* [55]. In addition, *Sasa veitchi* extract supplementation increased liver SOD activity in goldfish (*Carassius auratus*) infected with *Aeromonas salmonicida* [56], thereby reducing *A. salmonicida* abundance.

*Shewanella*, a conditional pathogen in fish, is widely found in the gut of marine, freshwater, and aquatic animals and damages the host intestinal immune system [57]. In the last two decades, *Shewanella* resistance to antibiotics has gained attention because several antibiotic genes have been identified on chromosomes associated or unassociated with mobile genetic elements (MGEs) related to resistance, making it an important vector for antibiotic resistance. *Shewanella* spp. abundance was significantly lower after quercetin treatment compared to the control (*p* < 0.01), which suggests that quercetin could be used as an antibiotic substitute to reduce the *Shewanella* risk to fish.

Reactive oxygen species (ROS) are signaling molecules and play a crucial role in the progression of inflammatory diseases and help clear pathogens. However, excess ROS levels can cause organ damage and kill normal cells [58], but antioxidant enzymes can counteract excess ROS [59]. Superoxide dismutase catalyzes the disproportionation of O_2_ to produce H_2_O_2_ [60], H_2_O_2_ is then converted to H_2_O by GSH-Px enzymes, and if antioxidant enzymes are inhibited resulting in H_2_O_2_ accumulation, cell membranes are damaged, and high levels of malondialdehyde (MDA) are released. The results from this study showed that the addition of quercetin increased the activities of hepatic antioxidant enzymes (SOD, POD, GST, GSH, and GSH-Px) and T-AOC levels compared with the CK group, thereby increasing the hepatic antioxidant capacity and scavenging free radicals in *O. potamophila*. These results are consistent with previous studies [61,62]. Moreover, quercetin protects zebrafish livers against TPT-induced damage by increasing the activities of antioxidant enzymes, reducing ROS levels, inhibiting apoptosis, and by reducing inflammation [63].

Anti-lipopolysaccharide factor (ALF) has strong antibacterial activity against Gram-negative R-type bacteria [64], and its expression significantly increased in both the 5 and 10 mg/L groups. The Toll-like receptor (TLR) signaling pathway, which is primarily expressed by innate immune cells, recognizes intestinal bacteria and their metabolites and is an essential element of innate immunity [65]. Toll-like receptor activation by pathogenic molecules derived from microbes, such as lipopolysaccharide (LPS), results in the activation of multiple intracellular signaling pathways and transcriptional events, which further induces the essential antimicrobial activity that initiates the body defense mechanism [66,67]. A related study showed that co-treatment with the flavonoid hesperetin significantly reduced inflammatory cytokine expression by improving Toll-like receptor-4-mediated expression of ionized calcium-binding adapter molecule 1/glial fibrillary acidic protein (Iba-1/GFAP) [68]. Quercetin also limits LPS-induced inflammation by inhibiting Src- and Syk-mediated phosphatidylinositol-3-kinase (PI3K)-(p85), tyrosine phosphorylation, and subsequent Toll-like receptor 4 (TLR4)/MyD88/PI3K complex formation (Endale et al., 2013) The TLR1, and MYD88 expression levels increased in the treated groups. In conjunction with previous findings, this suggests that quercetin can boost immunity by regulating the Toll pathway and by activating the expression of antimicrobial peptides. Tight junction proteins are an important component of the intestinal mucosal mechanical barrier [69]. OCLN and ZO-1 can form a stable association, which is important for maintaining the integrity of the intestinal mucosal barrier [70]. Previous studies have reported enhanced mRNA expression of ZO-1 and OCLN after the administration of flavonoids [71,72], and phenolic compounds influence the ability of these proteins to bind tightly and improve barrier integrity [73]. This study found that adding quercetin to the gut raised the mRNA expression of OCLN and ZO-1, indicating that quercetin promoted the protective action of the intestinal barrier. TNF- and IL-1, two pro-inflammatory cytokines, are indicators for immunomodulatory molecules that are produced early in fish infection and are crucial when attempting to control inflammation [74]. In comparison to the control group, IL-1 was elevated in *O. potamophila* livers following 5 mg/L and 10 mg/L quercetin treatment. IL-1β was upregulated in the liver after exposure to quercetin. Upregulation of IL-1β gene expression has also been observed in carp fed with glutamate [75] or spirulina [76]. Apoptosis, also known as programmed cell death, happens when certain harmful chemicals trigger cell death and is crucial for preserving body equilibrium [77]. Caspase-3 is an important component of the apoptosis pathway [78]. There were no significant differences in the changes of Caspase-3 genes, and the effects of quercetin on apoptosis-related genes need to be further investigated. Overall, this research demonstrates that quercetin can preserve immunological homeostasis while preserving intestinal barrier integrity. Quercetin also improves barrier function by decreasing the relative presence of potentially hazardous bacteria (*Plesiomonas, Aeromonas*, and *Shewanella*) in the gut microbiota. The COG and KEGG database analyses, which were used to predict the functions of intestinal bacteria, also showed that quercetin made improvements to the composition of the intestinal microbiota.

## 5. Conclusions

Illumina next-generation sequencing was used to analyze the different physiological states of *O. potamophila* during quercetin exposure. An analysis of the common intestinal microflora found in the samples exposed to three concentrations of quercetin showed that probiotics and pathogens were present in *O. potamophila*. In addition, antioxidant enzymes, such as SOD, POD, GST, GSH, GSH-Px, and T-AOC were elevated after treatment. In summary, this study increases our knowledge about the responses of fish gut microbes to quercetin.

## Figures and Tables

**Table 1:** Primer names and sequences.

Primer Name	Sequence (5′ to 3′)
ALF1 F	CTGCTTCCTGCATGGACCTA
ALF1 R	CACTGAACTCTCCCGCTTGT
TLR1 F	CAAAACGTCCAAAGTGCGGT
TLR1 R	AGTCTCCACGATTGCCAGTG
TLR2 F	ACTCTGAGCCGCTGATAACG
TLR2 R	CCAGGTTCATCCGAGTCACC
MYD88 F	CGCTGAGCTCATGGGATTCT
MYD88 R	TCAGGGCTCGTCCAGTATGA
OCLN F	TCAGGGCTCGTCCAGTATGA
OCLN R	AACCAGGAAGCCACAAACCA
ZO-1 F	CGCAGGTAGACGGCTCTAAA
ZO-1 R	GAGCTGATTGGTCTCCGTCC
TNF F	ACCGTAACAACGTGCCTCAT
TNF R	ACTGGCTTTCAGGACTGTCG
IL1B F	CCGAGGCACACTTGAAGACT
IL1B R	GAGTCCGGCTCACACATCTC
Caspase-3 F	AGAGCGTCATATACGGCACG
Caspase-3 R	CCTCTATGTCTTCGTCCGGC
Beta-actin F	CTCTTCCAGCCATCCTTCCT
Beta-actin R	TCAGGTGGGGCAATGATCTT

## Data Availability

The data was uploaded and the BioProject accession number is PRJNA853893.
